# Superficial Retinal Vascular Network Morphology and Sectoral RNFL Thickness in Children with a History of Bilateral Congenital Cataract Surgery: An Exploratory OCT/OCTA Study

**DOI:** 10.3390/jcm15135320

**Published:** 2026-07-07

**Authors:** Mehmet Omer Kiristioglu, Ahmet Tuncer Ozmen, Meral Yildiz, Ahmet Akcan, Esin Sogutlu Sari, Mehmet Baykara

**Affiliations:** 1 Department of Ophthalmology, Faculty of Medicine, Bursa Uludağ University, 16059 Bursa, Turkey; meraly@uludag.edu.tr (M.Y.); akcanahmet_50@hotmail.com (A.A.); esinsari@uludag.edu.tr (E.S.S.); mbaykara@uludag.edu.tr (M.B.); 2 Private Practice, 16059 Bursa, Turkey; ahmetozmen@yahoo.com

**Keywords:** congenital cataract, optical coherence tomography angiography, retinal vascular morphology, amblyopia, retinal nerve fiber layer

## Abstract

**Background**: Whether congenital cataract-related visual deprivation and subsequent rehabilitation are associated with retinal structural and vascular network differences remains unclear. This study evaluated superficial retinal vascular network descriptors and retinal nerve fiber layer (RNFL) thickness in children after bilateral congenital cataract extraction and secondary intraocular lens implantation. **Methods**: Age-matched children served as controls. Participants underwent spectral-domain optical coherence tomography (OCT) and 6 × 6 mm optical coherence tomography angiography (OCTA). Metrics were magnification-corrected, and comparisons used generalized estimating equation models adjusted for age, eye side, axial length, and spherical equivalent. **Results**: Eighteen pseudophakic children (36 eyes; median age, 8 years) and 17 controls (34 eyes; median age, 9 years) were included. In adjusted models, macular mean vessel diameter was higher in pseudophakic eyes (β = 11.16 µm; *p* = 0.003), as was macular mean tortuosity (β = 0.032; *p* = 0.001). Branchpoint density was lower in direction but not independently significant (*p* = 0.125). Choroidal thickness, choroidal vascularity index, and foveal avascular zone area did not differ significantly. Temporal RNFL thickness was greater in pseudophakic eyes (β = 13.37 µm; *p* = 0.007); other RNFL parameters were not significant. **Conclusions**: These findings suggest exploratory differences in superficial vascular network morphology and temporal RNFL thickness. However, because the groups differed clinically in best-corrected visual acuity, refractive status, and axial length, residual confounding cannot be excluded despite magnification correction and adjusted modeling.

## 1. Introduction

Congenital or infantile cataracts—lens opacities present within the first year of life—are uncommon but remain a major cause of childhood blindness worldwide [1,2]. Because the first years of life are critical for visual development, lens opacity during this period can disrupt normal visual input, leading to permanent changes in ocular growth and neural visual pathways if left untreated [3,4]. Early cataract removal, followed by optical correction and amblyopia therapy, is essential to optimize visual potential, with secondary intraocular lens (IOL) implantation commonly performed once the eye has grown sufficiently to ensure refractive stability [5].

While structural outcomes after pediatric cataract surgery have been examined in unilateral and bilateral cases, most imaging studies have focused on macular thickness or retinal nerve fiber layer (RNFL) profiles, with limited attention to the retinal microvasculature—particularly in bilaterally operated children. Amblyopia-related macular changes have been reported in both anisometropic and strabismic cases, but these findings may not fully represent the unique effects of bilateral visual deprivation caused by congenital cataracts [6,7].

Fourier-domain optical coherence tomography (OCT) has revealed that even when macular architecture appears grossly normal after surgery, subtle postoperative thickening can persist [8]. Optical coherence tomography angiography (OCTA) extends this structural assessment by visualizing the retinal and peripapillary microvasculature in a depth-resolved, non-invasive manner. Although OCTA has been utilized in pediatric conditions such as anisometropic amblyopia, retinopathy of prematurity, and uveitis, to our knowledge, it has not been systematically applied to evaluate eyes following bilateral congenital cataract extraction and secondary IOL implantation.

This study addresses this gap by combining OCTA with OCTAVA-based quantitative analysis to characterize superficial retinal vascular network morphology in children with a history of bilateral congenital cataract extraction and secondary IOL implantation. By evaluating OCTA-derived vascular network descriptors alongside RNFL thickness, we aimed to explore whether this rare postoperative pediatric population shows retinal structural or vascular morphology differences that may not be captured by conventional OCT thickness metrics alone.

## 2. Materials and Methods

### 2.1. Study Design and Ethics

This retrospective, cross-sectional comparative study included pediatric patients who had undergone bilateral congenital cataract extraction followed by secondary IOL implantation. In this retrospective study, imaging and clinical data were retrieved from postoperative visits conducted between January 2024 and February 2025 at Bursa Uludağ University Faculty of Medicine, with all included examinations performed ≥12 months after surgery. The study was approved by the Institutional Review Board of Bursa Uludağ University Faculty of Medicine (Ethics approval no: 2025/8-17; decision no: 621-8/17, dated 30 April 2025) and conducted in accordance with the Declaration of Helsinki.

### 2.2. Participants

Eligible patients had a confirmed diagnosis of bilateral congenital cataract, birth after 37 weeks’ gestation, primary bilateral cataract extraction before 1 year of age, subsequent secondary IOL implantation during early childhood, and at least 1 year of postoperative follow-up after IOL implantation. All imaging was obtained at ages 4–16 years, and best-corrected visual acuity (BCVA) had to be at least 20/400 in each operated eye. Initially, a total of 42 patients were recruited (21 in the study group and 21 in the control group); however, due to insufficient OCTA image quality, poor cooperation, and missing data, 3 patients from the study group and 4 patients from the control group were excluded, leaving 18 and 17 patients, respectively, for the final analysis.

Healthy, age-matched children without any known systemic or ocular diseases were recruited as controls. In addition to being free of ocular/systemic pathology, the same exclusion criteria applied to the study group were also applied to the controls to ensure comparability. Between-group differences in axial length (AL) and refractive status were modeled in all GEE analyses (covariates: age, AL, SE).

The same exclusion criteria were applied to both the pseudophakic study group and the healthy control group. Children were excluded if any of the following were present:

- Anterior segment abnormality other than pseudophakia in the study group.
- Corneal pathology, including corneal edema or opacity affecting image quality.
- History of ocular trauma, other than cataract surgery in the study group.
- Current or previous aphakic glaucoma.
- Retinal or optic nerve pathology, including retinal dystrophy, optic atrophy, coloboma, tilted disc, myopic crescent, or clinically visible optic disc drusen.
- History of pars plana vitrectomy or glaucoma surgery.
- History of retinopathy of prematurity.
- BCVA worse than 20/400, nystagmus, manifest strabismus, or poor fixation preventing reliable OCT/OCTA imaging.
- High refractive or biometric outliers, defined as an absolute spherical or cylindrical refractive component > 6.00 D, AL outside the 19–24 mm range, or interocular AL difference > 2 mm. This criterion was based on the individual spherical and cylindrical refractive components, not on calculated SE.
- Systemic, neurological, or metabolic disease known to affect ocular development.
- Poor-quality OCTA imaging, defined as IQS < 40, uncorrectable motion artifacts, or uncorrectable segmentation errors.

### 2.3. Surgical Technique

All cataract extractions were performed under general anesthesia by the same surgical team of experienced pediatric and anterior segment surgeons using a standardized technique. Limbal incisions were made, the anterior capsule was stained with trypan blue, and a 5-mm continuous curvilinear capsulorrhexis was created. Lens aspiration was performed using bimanual irrigation/aspiration. Posterior capsulorrhexis and limited anterior vitrectomy were conducted with triamcinolone-assisted (Sinakort-A 40 mg/mL injectable suspension; İ.E. Ulagay İlaç Sanayii Türk A.Ş., Istanbul, Türkiye)vitreous visualization.

Secondary IOL implantation with an Alcon SN60AT lens (Alcon Laboratories, Inc., Fort Worth, TX, USA) was performed after 3 years of age, at a median implantation age of 54.5 months (IQR, 46.3–77.3). The IOL was placed in the ciliary sulcus with optic capture (Table 1). Postoperative management included optical correction with spectacles or contact lenses and alternating occlusion therapy of the fellow eye for half of waking hours. Prescriptions were updated if the refractive error changed >1.00 D during 3-month follow-up visits.

### 2.4. Ophthalmic Examination

All participants underwent a standardized ophthalmic evaluation, including BCVA measurement, intraocular pressure assessment, slit-lamp biomicroscopy, tear film evaluation, dilated fundus examination, and ocular biometry using the Lenstar LS 900 (Haag-Streit AG, Köniz, Switzerland). BCVA was recorded in decimal notation in the clinical records and converted to logMAR for statistical analyses. For all participants, the BCVA values used in the analysis were obtained at the OCT/OCTA imaging visit or, when same-day BCVA was not available, from the closest contemporaneous ophthalmic examination. Amblyopia treatment duration was determined from chart documentation of prescribed occlusion therapy during follow-up; actual treatment compliance could not be reliably quantified retrospectively.

### 2.5. Imaging Protocol

Retinal and optic nerve head microvasculature were imaged with the DRI OCT Triton (Topcon Healthcare, Inc., Oakland, NJ, USA). Each eye underwent both a 6 × 6 mm macular scan and a 6 × 6 mm optic nerve head scan. Only scans with IQS > 40 were analyzed. The refractive error was entered into the device software prior to acquisition to minimize optical aberrations. All imaging was performed between 12:00 and 16:00 by the same examiner to reduce diurnal variation effects.

Because only refractive error, and not AL, was entered into IMAGEnet at acquisition, device-level lateral scaling was not applied. Therefore, metrics derived from physical dimensions were corrected offline using Bennett–Littmann scaling with a reference AL of 23.82 mm. Length-type metrics, including mean vessel diameter (MD), median vessel diameter, and total vessel length (TVL), were multiplied by the scaling factor [(AL − 1.82)/(23.82 − 1.82)], whereas area-type metrics were multiplied by the square of this factor [9]. Percentage-based area measures, such as vessel area density (VAD), were not altered. To avoid double correction, exported metadata were inspected; when pixel size did not vary across eyes, external scaling was applied. Magnification-corrected values were used for the primary analyses, and sensitivity analyses repeated without magnification correction did not materially change the inferences.

Analyses were performed exclusively on the superficial capillary plexus (SCP). The SCP slab was defined per device default from the internal limiting membrane (ILM) to the IPL/INL boundary, and SCP en-face angiograms were generated using maximum-intensity projection within the slab. For peripapillary analyses, the radial peripapillary capillaries slab was used (from the ILM to the lower boundary of the RNFL). The deep capillary plexus (DCP) and choriocapillaris were excluded from the analysis, as the deeper layers in pediatric patients are highly susceptible to projection artifacts from the superficial vessels and motion artifacts caused by fixation difficulties [10].

All automatic segmentations were reviewed by a grader masked to group allocation; minor boundary misalignments were manually corrected. Scans with uncorrectable segmentation errors were planned to be excluded from the analysis; however, all 70 scans (36 in the study group and 34 in the control group) that passed the initial image quality criteria had correctable segmentations and were successfully included in the final quantitative analysis.

### 2.6. Optical Coherence Tomography Assessments

Peripapillary RNFL thickness and choroidal assessments were performed using the Heidelberg Spectralis OCT (Heidelberg Engineering, Heidelberg, Germany). For RNFL evaluation, a standard peripapillary circular scan (3.4 mm diameter) centered on the optic disc was obtained. Global and sectoral RNFL thicknesses were calculated automatically by the device’s software. All RNFL segmentations were meticulously reviewed and, if necessary, manually corrected by an experienced ophthalmologist blinded to the participants’ clinical status.

Subfoveal choroidal thickness (CT) was measured perpendicularly from the retinal pigment epithelium to the sclerochoroidal junction in four quadrants (0.5 mm and 1.5 mm from the fovea) using enhanced depth imaging (EDI) mode with a 9.0 mm seven-line scan centered on the foveola. The auto-rescan function was used for consistency.

Choroidal vascularity index (CVI) was calculated from a 1.5 mm wide subfoveal region of interest using ImageJ software (v2.0.0, NIH, Bethesda, MD, USA). Three large choroidal vessels (>100 µm) were sampled to determine average reflectivity, after which images were binarized using Niblack’s thresholding method, converted to RGB, and the luminal area was segmented with the Threshold tool. CVI was computed as the ratio of luminal to total choroidal area, following previously described protocols [11,12]. All CT and CVI measurements were performed by a masked, experienced ophthalmologist blinded to group allocation.

### 2.7. OCTA Quantitative Analysis

Motion and projection artifacts were suppressed using the device’s built-in software, which incorporates active eye tracking and projection-artifact removal. For each scan, an image quality score (IQS; 0–100) was generated by the system, and scans with an IQS < 40 were excluded. OCTA scans were then exported from the DRI OCT Triton using IMAGEnet 6 software (v1.23; Topcon Healthcare, Inc., Oakland, NJ, USA) and analyzed with MATLAB (R2024a; MathWorks, Inc., Natick, MA, USA) integrated with the OCTAVA platform. Magnification correction was performed as described above.

Binarization and skeletonization were applied to each image before quantitative measurement. Vessel diameters were calculated from the distance transform of the binarized image, branchpoints were detected automatically and verified manually, tortuosity was calculated for each segment and averaged, and fractal dimension (FD) was computed via box-counting analysis. All OCTA analyses were performed by an observer masked to the participants’ clinical status. (A detailed description and the potential biological relevance of each evaluated metric are summarized in Supplementary Table S1) (Adapted from Untracht et al., 2021 [13]).

The foveal avascular zone (FAZ) measurements were performed using the Kanno–Saitama Macro (KSM), an automated ImageJ-based macro for FAZ extraction, in ImageJ software version 1.54p (National Institutes of Health, Bethesda, MD, USA) [14]. This method compensates for disruptions in the perifoveal capillary ring to achieve accurate delineation of the FAZ. Briefly, en face OCTA images from the SCP slab were downsized to 800 × 800 pixels to smooth boundary detection, followed by image binarization and skeletonization. Among various binarization algorithms, the Li method was applied [15]. Capillary ring discontinuities were corrected by iterative dilation and erosion, optimized through preliminary trials. Finally, the images were restored to their original resolution, and the FAZ area was extracted with a 2-pixel enlargement to ensure boundary accuracy. All images were processed using the same ImageJ macro in a blinded fashion to maintain objectivity and consistency.

### 2.8. Statistical Analysis

Statistical analyses were performed using the Statistical Package for the Social Sciences (SPSS) version 28.0 for Mac (SPSS Inc., Chicago, IL, USA). GraphPad Prism version 11.0.2 for Windows (GraphPad Software, Boston, MA, USA) was used for graphical visualization. A two-sided *p* value < 0.05 was considered statistically significant. The normality of data distribution was assessed using the Shapiro–Wilk test, with *p* < 0.05 indicating non-normal distribution. For parameters that did not follow a normal distribution, unadjusted comparisons between the study and control groups were performed using the Mann–Whitney U test. Although several variables were summarized as medians and interquartile ranges and compared using Mann–Whitney U tests in unadjusted analyses, GEE models with a normal distribution and identity link were used to estimate adjusted mean differences for continuous OCT/OCTA and RNFL outcomes. Robust sandwich standard errors were used to reduce sensitivity to distributional assumptions.

Primary OCTA endpoints were prespecified as macular mean vessel diameter (MD), mean tortuosity (MT), and branchpoint density (BD), based on the hypothesis of altered superficial retinal vascular network morphology in children with a history of bilateral congenital cataract surgery. Global and sectoral RNFL thicknesses were evaluated as exploratory structural OCT outcomes rather than as confirmatory primary endpoints, because previous OCT studies in amblyopia have reported inconsistent RNFL findings across sectors and amblyopia subtypes [16,17,18]. The primary OCTA endpoints were analyzed using generalized estimating equation (GEE) models with a normal distribution and identity link. Participant ID was specified as the clustering variable to account for inter-eye correlation, and an exchangeable working correlation structure with robust sandwich standard errors was used. Fully adjusted models included group, age, eye side, AL, and spherical equivalent (SE). Sensitivity analyses were performed using three covariate structures: age and eye side plus AL alone, age and eye side plus SE alone, and age and eye side plus both AL and SE. Group-by-eye side interactions were explored for the primary OCTA endpoints.

Because this was a retrospective exploratory study involving a rare pediatric postoperative population, no formal a priori sample size calculation was performed. All eligible children with bilateral congenital cataract surgery, secondary IOL implantation, and analyzable OCT/OCTA imaging during the study period were included. Therefore, the study should be considered hypothesis-generating rather than confirmatory. Other OCTA and RNFL parameters were considered exploratory secondary outcomes. Given the exploratory design and limited sample size, no formal adjustment for multiple comparisons was applied to secondary outcomes; therefore, these findings were interpreted with caution and evaluated together with effect sizes and 95% confidence intervals.

## 3. Results

A total of 18 children (8 girls, 10 boys) were included in the pseudophakic study group, and 17 healthy children (8 girls, 9 boys) were included in the control group. The median age at OCT/OCTA imaging was 8 years (range, 5–16 years) in the study group and 9 years (range, 6–16 years) in the control group. All operated children had undergone bilateral cataract extraction before one year of age. Baseline clinical, surgical, biometric, and imaging characteristics are summarized in Table 1.

At the time of OCT/OCTA imaging or the closest contemporaneous ophthalmic examination, the median BCVA in pseudophakic eyes was 0.22 logMAR in both right and left eyes (decimal equivalent, 0.6), with a range of 0.00–1.00 logMAR. This was significantly worse than in control eyes, where the median BCVA was 0.00 logMAR in both eyes (decimal equivalent, 1.0), with a range of 0.00–0.10 logMAR for both right and left eyes (*p* < 0.05 for both comparisons).

Refractive assessment revealed a more myopic profile in the pseudophakic study group. The median SE was −2.63 D (range, −6.12 to +0.50 D) in right eyes and −2.75 D (range, −5.88 to +2.38 D) in left eyes. In contrast, control eyes showed a hyperopic tendency, with median SE values of +0.94 D (range, −0.68 to +4.62 D) and +1.25 D (range, 0 to +6.00 D) in right and left eyes, respectively (*p* < 0.05 for both comparisons). AL was significantly greater in the pseudophakic group [right eyes: 21.81 mm (range, 19.10–23.84 mm); left eyes: 21.66 mm (range, 19.44–23.82 mm)] than in controls [right eyes: 20.44 mm (range, 19.02–22.47 mm); left eyes: 20.48 mm (range, 19.04–22.15 mm)] (right eyes, *p* = 0.009; left eyes, *p* = 0.021).

There were no statistically significant differences between groups in CT (right eyes: 384.5 µm vs. 373 µm, *p* = 0.264; left eyes: 373 µm vs. 363 µm, *p* = 0.052) or CVI (right eyes: 37.73% vs. 32.68%, *p* = 0.055; left eyes: 35.80% vs. 34.93%, *p* = 0.710).

**Figure 1:**
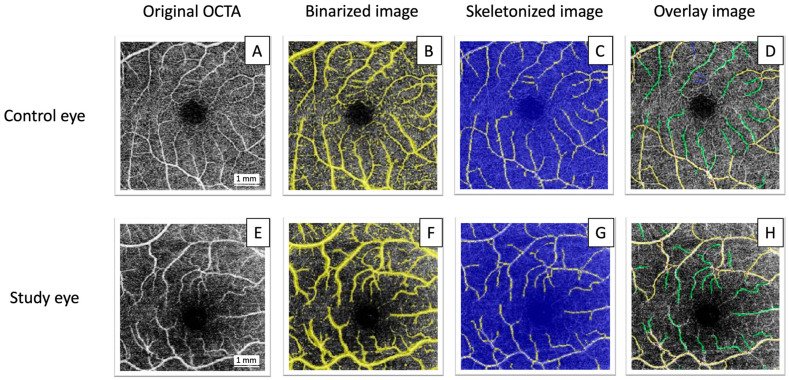
Representative 6 × 6 mm en-face OCTA images from a control eye and a study eye are shown. The original OCTA images were processed by binarization and skeletonization for quantitative vascular analysis. Panels (**A**–**D**) show the original OCTA, binarized, skeletonized, and overlay images from a representative control eye, respectively. Panels (**E**–**H**) show the corresponding images from a representative study eye. Scale bar: 1 mm.

Unadjusted eye-specific OCTA comparisons showed differences in selected superficial retinal vascular network descriptors. Macular MD was higher in the pseudophakic group in both eyes (right eyes: 116 vs. 107 µm, *p* = 0.049; left eyes: 112 vs. 110 µm, *p* = 0.044). Macular MT did not differ significantly in right eyes (1.15 vs. 1.14, *p* = 0.126), whereas it was higher in pseudophakic left eyes (1.15 vs. 1.12, *p* = 0.048). Macular BD was lower in pseudophakic eyes in both eyes in unadjusted comparisons (right eyes: 1.029 vs. 1.290 nodes/mm, *p* = 0.018; left eyes: 1.033 vs. 1.110 nodes/mm, *p* = 0.036). Other exploratory OCTA parameters, including VAD, VLD, TVL, and FD, did not show consistent significant between-group differences. Peripapillary OCTA metrics were generally comparable between groups, except for MT, which reached statistical significance in both eyes but showed directionally inconsistent differences; therefore, peripapillary MT was interpreted cautiously. Representative OCTA images and the corresponding binarized and skeletonized images are shown in Figure 1. Detailed unadjusted macular and peripapillary OCTA metrics are presented in Table 2.

**Figure 2:**
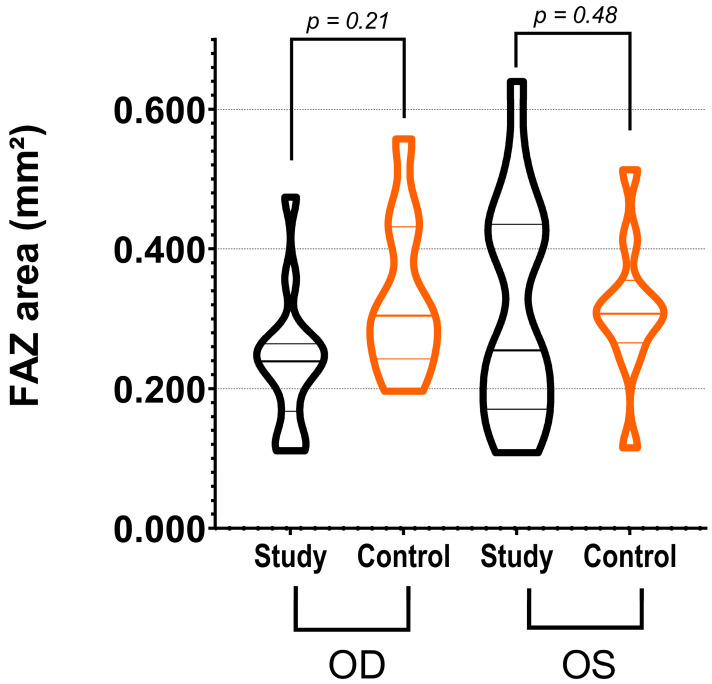
Foveal avascular zone (FAZ) area in pseudophakic study eyes and healthy control eyes. FAZ area is reported in mm^2^. Although FAZ area was numerically smaller in the study group in both right and left eyes, the differences were not statistically significant.

The median FAZ area was numerically smaller in the pseudophakic group than in controls in both eyes. In right eyes, the median FAZ area was 0.240 mm^2^ (IQR, 0.106) in the pseudophakic group and 0.303 mm^2^ (IQR, 0.220) in controls. In left eyes, the corresponding values were 0.255 mm^2^ (IQR, 0.262) and 0.305 mm^2^ (IQR, 0.114), respectively. However, these differences were not statistically significant (right eyes: U = 46.0, *p* = 0.210; left eyes: U = 59.0, *p* = 0.480) (Figure 2). Macular and peripapillary OCTA image quality scores were analyzed separately. There was no significant difference between the groups in either macular or peripapillary OCTA IQS (*p* = 0.441 and *p* = 0.071, respectively).

Sectoral RNFL analysis showed numerically greater thickness in several sectors of pseudophakic eyes. However, in fully adjusted models, only temporal RNFL thickness remained significantly greater in pseudophakic eyes (β = 13.37 µm; 95% CI, 3.61 to 23.14; *p* = 0.007). Global RNFL thickness and the remaining sectoral RNFL parameters were not significantly different in fully adjusted models. Exact sectoral RNFL values, variability measures, and adjusted GEE comparisons are provided in Table 3.

In fully adjusted GEE models including age, eye side, AL, and SE, macular MD remained significantly higher in pseudophakic eyes than in control eyes (β = 11.16 µm; 95% CI, 3.79 to 18.53; *p* = 0.003). Macular MT was also higher in pseudophakic eyes after full adjustment (β = 0.032; 95% CI, 0.014 to 0.051; *p* = 0.001), although the magnitude of this difference was small. Macular BD was lower in pseudophakic eyes, but this difference did not reach statistical significance in the fully adjusted model (β = −0.139; 95% CI, −0.316 to 0.039; *p* = 0.125). Group-by-eye-side interaction terms were not significant for the primary OCTA outcomes, including macular MD, MT, and BD (*p* = 0.995, *p* = 0.487, and *p* = 0.285, respectively). Sensitivity analyses using AL alone, SE alone, and both covariates together showed that the MD and MT findings remained significant across model specifications, whereas BD did not remain independently significant. The fully adjusted GEE and sensitivity analysis results are presented in Table 4.

## 4. Discussion

In this retrospective cross-sectional cohort of children who underwent bilateral congenital cataract extraction followed by secondary IOL implantation, we observed differences in selected OCTA-derived superficial retinal vascular network descriptors and sectoral RNFL thickness compared with healthy controls. After adjustment for age, eye side, AL, and SE, macular MD and MT remained higher in pseudophakic eyes, whereas BD was lower in direction but did not remain independently significant. Among exploratory structural OCT outcomes, only temporal RNFL thickness remained significantly greater after full adjustment. These findings suggest that children with a history of bilateral congenital cataract surgery may show persistent differences in superficial retinal vascular network morphology and sectoral RNFL profile; however, the cross-sectional design precludes causal inference regarding whether these differences reflect congenital ocular development, deprivation amblyopia, postoperative ocular growth, optical factors, or surgery-related effects.

To our knowledge, previous studies have not systematically applied OCTA with OCTAVA-based quantitative vascular network analysis to children after bilateral congenital cataract surgery and secondary IOL implantation. Previous imaging studies in pediatric cataract and amblyopia have mainly focused on macular thickness, RNFL thickness, or conventional OCT-derived structural metrics. By combining OCTA-derived vascular network descriptors with sectoral RNFL measurements, the present study provides an exploratory framework for assessing retinal morphology beyond conventional thickness-based outcomes in this rare postoperative pediatric population.

The RNFL findings deserve cautious interpretation. In deprivation amblyopia and congenital cataract, reduced early visual input might be expected to impair visual pathway development, and several studies have reported RNFL thinning in amblyopic eyes, particularly in unilateral cases or specific quadrants [19,20,21]. In contrast, we observed greater temporal RNFL thickness in pseudophakic eyes, and this finding remained significant after adjustment for AL and refractive status. This is noteworthy because longer AL is generally associated with lower measured RNFL thickness and altered optic disc parameters [22,23]. However, sectoral RNFL thickness can be influenced by scan-circle magnification, optic disc configuration, segmentation quality, pediatric normative variability, amblyopia subtype, and ocular growth. Therefore, the temporal RNFL finding should be regarded as an exploratory structural association rather than evidence of a specific compensatory or postoperative mechanism.

Several explanations may account for the apparent temporal RNFL difference. Amblyopia-related alterations in retinal maturation have been reported in both structural OCT and RNFL studies, although the direction and sectoral distribution of these findings vary across anisometropic, strabismic, and deprivation amblyopia [6,16,17,24,25]. Park et al. also reported increased thickness in several retinal layers in amblyopic eyes, supporting the concept that amblyopia may affect multiple retinal structures rather than the RNFL alone [26]. In our cohort, bilateral congenital cataract, residual amblyopia, optical correction, occlusion therapy, secondary IOL implantation, and postoperative ocular growth may all have contributed. Because reliable OCT imaging is rarely feasible during infancy and early postoperative follow-up, the temporal course of RNFL development in these eyes remains unclear. Longitudinal studies with preoperative, early postoperative, and long-term imaging are needed to determine whether the observed temporal RNFL difference represents altered development, treatment-related change, measurement-related variation, or residual confounding.

A transient surgical effect on RNFL thickness is another theoretical consideration. Dada et al. described postoperative RNFL thickening after adult cataract surgery, although this was measured using scanning laser polarimetry at week 4 and likely reflected an early postoperative phenomenon [27]. In contrast, our participants were imaged years after pediatric cataract surgery using spectral-domain OCT, making a direct parallel unlikely. Cataract-related media opacity may also lead to underestimation of RNFL measurements, but this explanation does not apply to our comparison because the operated eyes were pseudophakic at imaging and the control eyes had clear lenses [28].

The OCTAVA-based analysis identified higher macular MD and MT in pseudophakic eyes after full adjustment. These findings may indicate altered superficial retinal vascular network morphology in eyes exposed to early bilateral visual deprivation and subsequent optical rehabilitation. However, these parameters should not be regarded as direct histologic markers of capillary remodeling. This caution is particularly relevant for MT, because the absolute between-group difference was small despite statistical significance and may partly reflect the sensitivity of tortuosity indices to image acquisition, segmentation, binarization, skeletonization, projection artifacts, and the inclusion of larger superficial vessels. Therefore, a statistically significant MT difference does not necessarily imply a biologically meaningful vascular alteration. Because large vessels were not masked, MD and MT represent composite descriptors of the entire superficial vascular network rather than capillary-specific measurements. The observed differences should therefore be interpreted cautiously as OCTA-derived vascular network morphology differences, not as definitive evidence of microvascular remodeling.

BD was lower in pseudophakic eyes in unadjusted eye-specific comparisons, but this difference did not remain statistically significant after simultaneous adjustment for AL and SE. Therefore, reduced branching should not be considered an independent primary finding of the present study. The attenuation of BD after adjustment suggests that refractive status, AL, sample size, and image-processing factors may partly influence this metric. Reduced capillary density and altered macular vascular parameters have been reported in amblyopia, but the present findings should be interpreted within the limits of OCTA-derived network analysis and the exploratory design of this study [29,30,31]. Although lower BD may theoretically indicate reduced vascular interconnectedness, as suggested in other optic nerve diseases such as glaucoma, our fully adjusted results do not support a definitive conclusion regarding reduced branching in this cohort [32].

Peripapillary OCTA findings were less consistent than macular findings. Most peripapillary metrics did not differ significantly between groups. Although peripapillary MT reached statistical significance in unadjusted eye-specific analyses, the direction of difference was inconsistent between right and left eyes; therefore, this finding was not emphasized as a biologically robust result. Overall, the macular SCP-derived MD and MT findings appear more coherent than the peripapillary OCTA findings in the present dataset.

FAZ area, CT, and CVI did not differ significantly between groups. Previous OCTA studies in amblyopia have reported inconsistent FAZ findings, with most studies showing no significant difference, whereas some have reported smaller FAZ area in amblyopic eyes, particularly in the SCP [29,33,34,35]. To date, FAZ morphology has not been specifically characterized in congenital cataract using this type of postoperative OCTA framework. In the present study, FAZ area was numerically smaller in pseudophakic eyes but did not reach statistical significance. Similarly, the absence of significant CT or CVI differences may reflect the heterogeneity of choroidal responses reported in amblyopia and pediatric cataract studies, as well as differences in AL, age at imaging, surgical timing, and treatment history [36,37,38,39,40,41].

AL and refractive status are central to the interpretation of the present findings. Pseudophakic eyes were more myopic and had longer AL than control eyes. These differences may influence OCT and OCTA measurements through ocular magnification, scan scaling, scan-circle geometry, and true anatomic changes related to axial elongation. Previous studies have shown that AL and refractive error can affect RNFL measurements, optic disc parameters, OCTA vessel-density metrics, and FAZ area, and that magnification correction may attenuate some of these relationships [22,23,42,43]. Peripapillary OCTA studies also suggest that the effect of AL on vessel density and perfusion density may be region-dependent, with different behavior in inner and outer peripapillary rings [44]. In addition, OCTAVA-derived metrics such as VAD, TVL, vessel length density, node count, BD, and FAZ area are influenced by segmentation boundaries, preprocessing, binarization, and skeletonization; therefore, they should be interpreted as quantitative vascular network descriptors rather than direct histologic measures of capillary remodeling [45].

In the present study, we attempted to mitigate AL and refractive-status effects by applying Bennett–Littmann magnification correction, excluding poor-quality scans, visually checking segmentation, and using GEE models adjusted for age, eye side, AL, and SE. Sensitivity analyses using AL alone, SE alone, and both covariates together showed that macular MD and MT remained significant across model specifications, whereas BD did not. Nevertheless, residual confounding cannot be excluded, especially given the limited sample size and the intrinsic clinical differences between pseudophakic eyes and healthy controls. Exploratory interaction analyses also suggested that the relationship between AL and OCTA metrics may differ between groups; therefore, AL-related effects should not be considered fully eliminated.

This study has several limitations. First, the retrospective cross-sectional design prevents determination of whether the observed differences predated surgery, developed during postoperative ocular growth, or were influenced by amblyopia treatment and optical rehabilitation. Second, the cohort was small because bilateral congenital cataract with analyzable pediatric OCT/OCTA imaging is rare; therefore, the analyses should be considered exploratory and hypothesis-generating. Third, the pseudophakic and control groups differed in BCVA, SE, and AL. Although these variables were addressed using adjusted GEE models and sensitivity analyses, residual confounding remains possible. Fourth, amblyopia severity and treatment compliance could not be quantified with sufficient precision from retrospective records. Fifth, pediatric OCTA is technically challenging; although scans with poor image quality, uncorrectable motion artifacts, or segmentation errors were excluded, subtle fixation instability or subclinical nystagmus could still have influenced the results [46]. Sixth, only the SCP and radial peripapillary capillary slab were analyzed; the deep capillary plexus was excluded because of the high susceptibility to projection and motion artifacts in pediatric imaging. Seventh, large vessels were not masked, so OCTAVA-derived morphometric metrics represent the entire superficial vascular network rather than isolated capillary-level morphology. Eighth, optic disc area, GCIPL/GCL thickness, and dedicated screening for subclinical optic disc drusen were not available for all participants. Finally, the absence of internal fellow-eye controls, inherent to the bilateral nature of the disease, necessitated comparison with external healthy controls.

In conclusion, this exploratory OCT/OCTA study suggests that children with a history of bilateral congenital cataract surgery and secondary IOL implantation may show higher macular MD and MT, together with greater temporal RNFL thickness, after adjustment for AL and refractive status. BD was lower in direction but did not remain independently significant after full adjustment. These findings should be interpreted as cross-sectional associations compatible with altered superficial retinal vascular network morphology, rather than as definitive evidence of surgery-induced or deprivation-induced vascular remodeling. Importantly, because the pseudophakic and control groups differed clinically in BCVA, SE, and AL, residual confounding cannot be excluded despite magnification correction and adjusted GEE modeling. Larger longitudinal studies with better refractive and biometric matching, capillary-specific OCTA analysis, GCIPL/GCL assessment, and functional correlations are needed to clarify the developmental and clinical significance of these findings.

## Figures and Tables

**Table 1:** Baseline clinical, surgical, and imaging characteristics of the study and control groups.

Variable	Pseudophakic Study Group	Control Group
Participant-level variables		
Age at OCT/OCTA imaging, years	8.0 (7.0–10.0)	9.0 (7.0–9.0)
Female/male, n (%)	8 (44.4)/10 (55.6)	8 (47.1)/9 (52.9)
Amblyopia treatment duration, months	82.0 (62.5–106.0)	Not applicable
Manifest strabismus at imaging	None	None
Nystagmus at imaging	None	None
Eye-level visual and biometric variables		
BCVA, logMAR	0.22 (0.10–0.49)	0.00 (0.00–0.00)
BCVA, decimal	0.60 (0.33–0.80)	1.00 (1.00–1.00)
Spherical equivalent, D	−2.62 (−3.25 to −1.75)	+1.13 (+0.50 to +1.87)
Axial length, mm	21.66 (21.20–22.90)	20.46 (19.76–21.45)
Eye-level surgical variables		
Age at cataract extraction, months	8.0 (4.0–11.0)	Not applicable
Age at secondary IOL implantation, months	54.5 (46.3–77.3)	Not applicable
Aphakia duration, months	47.5 (35.8–70.3)	Not applicable
Cataract morphology		
Lamellar	12 (33.3)	Not applicable
Total	7 (19.4)	Not applicable
Posterior polar	4 (11.1)	Not applicable
Zonular	3 (8.3)	Not applicable
Cortical	2 (5.6)	Not applicable
Blue-dot + lamellar	2 (5.6)	Not applicable
Sutural	2 (5.6)	Not applicable
Anterior subcapsular	2 (5.6)	Not applicable
Cerulean	2 (5.6)	Not applicable
IOL location		
Ciliary sulcus with optic capture	36 (100.0)	Not applicable
OCTA image quality score		
Macular OCTA IQS	72.0 (67.0–75.3)	75.5 (72.3–78.0)
Peripapillary OCTA IQS	71.5 (67.8–75.0)	73.0 (72.0–76.0)

Values are presented as median (interquartile range) or n (%). Aphakia duration was calculated as the interval between cataract extraction and secondary IOL implantation. IQS refers to the device-generated OCTA image quality score for macular and peripapillary angiography scans. Although spherical equivalent values are reported descriptively, the exclusion criterion was based on the absolute spherical or cylindrical components, not spherical equivalent. Therefore, spherical equivalent could slightly exceed ±6.00 D without violating the cutoff. BCVA, best-corrected visual acuity; D, diopters; IOL, intraocular lens; IQS, image quality score; OCT, optical coherence tomography; OCTA, optical coherence tomography angiography.

**Table 2:** Eye-specific unadjusted comparison of macular and peripapillary OCTA metrics between study and control groups.

Region	Parameter (Units)	Right Eye			Left Eye		
		Study Group	Control Group	Unadjusted *p* Value	Study Group	Control Group	Unadjusted *p* Value
Macula	VAD (%)	33.24 (29.53–39.65)	35.87 (33.45–36.33)	0.052	33.45 (27.59–35.98)	33.91 (32.0–35.33)	0.739
	VLD (%)	4.3 (3.22–4.64)	4.66 (2.22–5.08)	0.506	4.17 (2.93–4.9)	4.08 (4.0–4.93)	0.925
	TVL (mm)	68.78 (51.47–74.19)	74.54 (35.55–81.26)	0.87	63.72 (46.82–74.54)	65.27 (64.07–78.87)	0.79
	MD (µm)	116.0 (95.0–124.0)	107.0 (101.0–129.0)	0.049	112.0 (101.0–137.0)	110.0 (102.0–131.0)	0.044
	MED (µm)	97.0 (90.0–122.0)	94.0 (86.0–117.0)	0.29	104.0 (77.0–111.0)	95.0 (90.0–131.0)	0.22
	BD (nodes/mm)	1.0294 (0.6–1.17)	1.29 (0.39–1.36)	**0** **.** **018**	1.033 (0.72–1.15)	1.11 (0.84–1.15)	**0** **.** **036**
	FD (no unit)	1.86 (1.85–1.86)	1.86 (1.85–1.86)	0.532	1.84 (1.79–1.86)	1.86 (1.85–1.86)	0.251
	MT (no unit)	1.15 (1.11–1.28)	1.14 (1.13–1.27)	0.126	1.15 (1.12–1.19)	1.12 (1.11–1.13)	**0** **.** **048**
Disc	VAD (%)	33.6 (31.09–35.97)	32.87 (30.12–34.8)	0.217	31.2 (23.7–35.4)	33.0 (32.5–35.1)	0.167
	VLD (%)	4.43 (3.96–4.81)	4.33 (3.5–4.57)	0.215	4.0 (2.42–4.63)	4.11 (3.9–5.1)	0.15
	TVL (mm)	70.92 (63.4–76.94)	69.25 (55.94–73.12)	0.229	64.07 (52.54–67.0)	65.7 (62.38–81.64)	0.228
	MD (µm)	116.0 (107.0–130.0)	115.0 (112.0–125.0)	0.797	120.0 (105.0–132.0)	118.0 (110.0–130.0)	0.691
	MED (µm)	110.0 (94.0–128.0)	105.0 (102.0–119.0)	0.438	112.0 (97.0–131.0)	111.0 (103.0–133.0)	0.608
	BD (nodes/mm)	1.01 (0.87–1.38)	0.99 (0.86–1.16)	0.652	0.991 (0.65–1.16)	0.99 (0.88–1.03)	0.277
	FD (no unit)	1.86 (1.85–1.86)	1.86 (1.86–1.86)	0.23	1.86 (1.85–1.86)	1.86 (1.85–1.86)	0.776
	MT (no unit)	1.16 (1.14–1.18)	1.15 (1.11–1.16)	**0** **.** **036**	1.14 (1.11–1.21)	1.19 (1.14–1.26)	**0** **.** **047**

Values are presented as median (interquartile range). Right and left eyes were analyzed separately. *p* values represent unadjusted between-group comparisons. Adjusted GEE results are provided in Table 4. Bold *p* values indicate statistical significance (*p* < 0.05). Abbreviations: BD: Branchpoint Density, FD: Fractal Dimension, MD: Mean Diameter, MED: Median Diameter, MT: Mean Tortuosity, TVL: Total Vessel Length, VAD: Vessel Area Density, VLD: Vessel Length Density.

**Table 3:** Sectoral RNFL thickness values and adjusted GEE comparisons.

RNFL Parameter	Pseudophakic Study Group, Median (IQR)	Control Group, Median (IQR)	Adjusted β (95% CI)	Adjusted *p* Value
Global RNFL, µm	110.0 (96.2–122.2)	94.0 (87.5–104.2)	12.22 (−6.90 to 31.35)	0.210
Temporal RNFL, µm	79.0 (73.0–85.0)	58.5 (57.8–78.2)	13.37 (3.61 to 23.14)	**0.007**
Superotemporal RNFL, µm	138.5 (128.2–157.0)	124.5 (94.0–136.2)	12.63 (−6.94 to 32.20)	0.206
Superonasal RNFL, µm	110.0 (93.5–122.0)	101.0 (76.2–120.0)	15.50 (−7.72 to 38.73)	0.191
Nasal RNFL, µm	80.0 (73.8–93.0)	80.0 (51.0–93.0)	−2.39 (−23.70 to 18.91)	0.826
Inferonasal RNFL, µm	114.0 (92.2–131.5)	110.0 (82.0–126.8)	−5.45 (−37.25 to 26.35)	0.737
Inferotemporal RNFL, µm	160.0 (140.5–165.8)	141.0 (112.8–154.2)	10.24 (−21.24 to 41.73)	0.524

Values are presented as median (interquartile range). Adjusted β coefficients indicate the mean difference for pseudophakic eyes versus control eyes. GEE models were adjusted for age, eye side, axial length, and spherical equivalent. Bold *p* values indicate statistical significance (*p* < 0.05). Descriptive RNFL values and adjusted models were calculated from all eyes. CI, confidence interval; GEE, generalized estimating equation; IQR, interquartile range; RNFL, retinal nerve fiber layer.

**Table 4:** Fully adjusted GEE models and sensitivity analyses for OCTA and RNFL outcomes.

Outcome	Model	β Coefficient	Robust SE	95% CI	*p* Value	Eyes, n	Participants, n
**Primary OCTA outcomes**							
Macular MD, µm	Fully adjusted	11,160	3760	3.79 to 18.53	**0.003**	70	35
Macular MT	Fully adjusted	0.032	0.009	0.014 to 0.051	**0.001**	70	35
Macular BD, nodes/mm	Fully adjusted	−0.139	0.091	−0.316 to 0.039	0.125	70	35
**Exploratory RNFL outcomes**							
Global RNFL, µm	Fully adjusted	12,220	9760	−6.90 to 31.35	0.210	70	35
Temporal RNFL, µm	Fully adjusted	13,370	4980	3.61 to 23.14	**0.007**	70	35
Superotemporal RNFL, µm	Fully adjusted	12,630	9980	−6.94 to 32.20	0.206	70	35
Superonasal RNFL, µm	Fully adjusted	15,500	11,850	−7.72 to 38.73	0.191	70	35
Nasal RNFL, µm	Fully adjusted	−2390	10,870	−23.70 to 18.91	0.826	70	35
Inferonasal RNFL, µm	Fully adjusted	−5450	16,220	−37.25 to 26.35	0.737	70	35
Inferotemporal RNFL, µm	Fully adjusted	10,240	16,060	−21.24 to 41.73	0.524	70	35
**Sensitivity analyses for primary OCTA outcomes**							
Macular MD, µm	Age + eye side + AL	9720	4830	0.25 to 19.20	**0.044**	70	35
Macular MD, µm	Age + eye side + SE	11,410	3170	5.19 to 17.63	**<0.001**	70	35
Macular MD, µm	Age + eye side + AL + SE	11,160	3760	3.79 to 18.53	**0.003**	70	35
Macular MT	Age + eye side + AL	0.041	0.012	0.018 to 0.064	**0.001**	70	35
Macular MT	Age + eye side + SE	0.027	0.009	0.010 to 0.043	**0.002**	70	35
Macular MT	Age + eye side + AL + SE	0.032	0.009	0.014 to 0.051	**0.001**	70	35
Macular BD, nodes/mm	Age + eye side + AL	−0.078	0.092	−0.259 to 0.102	0.393	70	35
Macular BD, nodes/mm	Age + eye side + SE	−0.144	0.082	−0.305 to 0.017	0.080	70	35
Macular BD, nodes/mm	Age + eye side + AL + SE	−0.139	0.090	−0.316 to 0.039	0.125	70	35

β coefficients indicate adjusted mean differences for pseudophakic eyes versus control eyes. These model-based estimates may differ from the unadjusted median comparisons reported in Table 2. Models used participant ID as the clustering variable, exchangeable working correlation, and robust sandwich standard errors. Fully adjusted models included group, age, eye side, AL, and SE. Sensitivity models included age and eye side plus axial length, spherical equivalent, or both. Differences in eyes/participants reflect complete-case availability. Group-by-eye-side interaction results are reported in the Results. Bold *p* values indicate statistical significance (*p* < 0.05). AL, axial length; BD, branchpoint density; CI, confidence interval; GEE, generalized estimating equation; MD, mean vessel diameter; MT, mean tortuosity; OCTA, optical coherence tomography angiography; RNFL, retinal nerve fiber layer; SE, spherical equivalent.

## Data Availability

The datasets used and/or analyzed during the current study are available from the corresponding author upon reasonable request.

## Supplementary Materials

The following supporting information can be downloaded at: https://www.mdpi.com/article/10.3390/jcm15135320/s1, Table S1: Adapted Summary of Quantitative Metrics for Microvascular Network Architecture in OCTA (Adapted from Untracht et al., 2021 [13]).

## Abbreviations

AL, axial length; BCVA, best-corrected visual acuity; BD, branchpoint density; CT, choroidal thickness; CVI, choroidal vascularity index; FAZ, foveal avascular zone; FD, fractal dimension; GEE, generalized estimating equations; IOL, intraocular lens; IQS, image quality score; MD, mean vessel diameter; MT, mean tortuosity; OCT, optical coherence tomography; OCTA, optical coherence tomography angiography; OCTAVA, optical coherence tomography angiography vascular analyzer; RNFL, retinal nerve fiber layer; SCP, superficial capillary plexus; SE, spherical equivalent; TVL, total vessel length; VAD, vessel area density.

## References

[1] Gilbert C., Foster A. (2001). Childhood blindness in the context of VISION 2020—The right to sight. Bull. World Health Organ..

[2] Sheeladevi S., Lawrenson J.G., Fielder A.R., Suttle C.M. (2016). Global prevalence of childhood cataract: A systematic review. Eye.

[3] Rabin J., Van Sluyters R.C., Malach R. (1981). Emmetropization: A vision-dependent phenomenon. Investig. Ophthalmol. Vis. Sci..

[4] Wallman J., Winawer J. (2004). Homeostasis of eye growth and the question of myopia. Neuron.

[5] Birch E.E., Stager D.R. (1996). The critical period for surgical treatment of dense congenital unilateral cataract. Investig. Ophthalmol. Vis. Sci..

[6] Huynh S.C., Samarawickrama C., Wang X.Y., Rochtchina E., Wong T.Y., Gole G.A., Rose K.A., Mitchell P. (2009). Macular and nerve fiber layer thickness in amblyopia: The Sydney Childhood Eye Study. Ophthalmology.

[7] Al-Haddad C.E., El Mollayess G.M., Mahfoud Z.R., Jaafar D.F., Bashshur Z.F. (2013). Macular ultrastructural features in amblyopia using high-definition optical coherence tomography. Br. J. Ophthalmol..

[8] Wang J., Smith H.A., Donaldson D.L., Haider K.M., Roberts G.J., Sprunger D.T., Neely D.E., Plager D.A. (2014). Macular structural characteristics in children with congenital and developmental cataracts. J. AAPOS.

[9] Bennett A.G., Rudnicka A.R., Edgar D.F. (1994). Improvements on Littmann’s method of determining the size of retinal features by fundus photography. Graefes Arch. Clin. Exp. Ophthalmol..

[10] Diao K., Huang X., Yao M., Li J., Fan F., Pan H., Yu J., Yang Y., Lu W., Lian H. (2023). Inter-examiner and intra-examiner reliability of optical coherence tomography angiography in vascular density measurement of retinal and choriocapillaris plexuses in healthy children aged 6–15 years. Front. Med..

[11] Sonoda S., Sakamoto T., Yamashita T., Uchino E., Kawano H., Yoshihara N., Terasaki H., Shirasawa M., Tomita M., Ishibashi T. (2015). Luminal and stromal areas of choroid determined by binarization method of optical coherence tomographic images. Am. J. Ophthalmol..

[12] Zhu Q., Zhao Q. (2022). Short-term effect of orthokeratology lens wear on choroidal blood flow in children with low and moderate myopia. Sci. Rep..

[13] Untracht G.R., Matos R.S., Dikaios N., Bapir M., Durrani A.K., Butsabong T., Campagnolo P., Sampson D.D., Heiss C., Sampson D.M. (2021). OCTAVA: An open-source toolbox for quantitative analysis of optical coherence tomography angiography images. PLoS ONE.

[14] Ishii H., Shoji T., Yoshikawa Y., Kanno J., Ibuki H., Shinoda K. (2019). Automated Measurement of the Foveal Avascular Zone in Swept-Source Optical Coherence Tomography Angiography Images. Transl. Vis. Sci. Technol..

[15] Li C., Tam P.K.-S. (1998). An iterative algorithm for minimum cross entropy thresholding. Pattern Recognit. Lett..

[16] Repka M.X., Kraker R.T., Tamkins S.M., Suh D.W., Sala N.A., Beck R.W., Pediatric Eye Disease Investigator G. (2009). Retinal nerve fiber layer thickness in amblyopic eyes. Am. J. Ophthalmol..

[17] Yen M.Y., Cheng C.Y., Wang A.G. (2004). Retinal nerve fiber layer thickness in unilateral amblyopia. Investig. Ophthalmol. Vis. Sci..

[18] Parikh R., Sachdeva V., Kekunnaya R., Rao B.V., Parikh S., Thomas R. (2022). Retinal nerve fiber layer thickness in amblyopia. Indian J. Ophthalmol..

[19] Maguire G.W., Smith E.L., Harwerth R.S., Crawford M.L. (1982). Optically induced anisometropia in kittens. Investig. Ophthalmol. Vis. Sci..

[20] von Noorden G.K., Crawford M.L., Levacy R.A. (1983). The lateral geniculate nucleus in human anisometropic amblyopia. Investig. Ophthalmol. Vis. Sci..

[21] Bansal P., Ram J., Sukhija J., Singh R., Gupta A. (2016). Retinal Nerve Fiber Layer and Macular Thickness Measurements in Children After Cataract Surgery Compared with Age-Matched Controls. Am. J. Ophthalmol..

[22] Savini G., Barboni P., Parisi V., Carbonelli M. (2012). The influence of axial length on retinal nerve fibre layer thickness and optic-disc size measurements by spectral-domain OCT. Br. J. Ophthalmol..

[23] Leung C.K., Mohamed S., Leung K.S., Cheung C.Y., Chan S.L., Cheng D.K., Lee A.K., Leung G.Y., Rao S.K., Lam D.S. (2006). Retinal nerve fiber layer measurements in myopia: An optical coherence tomography study. Investig. Ophthalmol. Vis. Sci..

[24] Baddini-Caramelli C., Hatanaka M., Polati M., Umino A.T., Susanna R. (2001). Thickness of the retinal nerve fiber layer in amblyopic and normal eyes: A scanning laser polarimetry study. J. AAPOS.

[25] Altintas O., Yuksel N., Ozkan B., Caglar Y. (2005). Thickness of the retinal nerve fiber layer, macular thickness, and macular volume in patients with strabismic amblyopia. J. Pediatr. Ophthalmol. Strabismus.

[26] Park K.A., Park D.Y., Oh S.Y. (2011). Analysis of spectral-domain optical coherence tomography measurements in amblyopia: A pilot study. Br. J. Ophthalmol..

[27] Dada T., Behera G., Agarwal A., Kumar S., Sihota R., Panda A. (2010). Effect of cataract surgery on retinal nerve fiber layer thickness parameters using scanning laser polarimetry (GDxVCC). Indian J. Ophthalmol..

[28] Kim N.R., Lee H., Lee E.S., Kim J.H., Hong S., Je Seong G., Kim C.Y. (2012). Influence of cataract on time domain and spectral domain optical coherence tomography retinal nerve fiber layer measurements. J. Glaucoma.

[29] Yilmaz I., Ocak O.B., Yilmaz B.S., Inal A., Gokyigit B., Taskapili M. (2017). Comparison of quantitative measurement of foveal avascular zone and macular vessel density in eyes of children with amblyopia and healthy controls: An optical coherence tomography angiography study. J. AAPOS.

[30] Hormel T.T., Wang J., Bailey S.T., Hwang T.S., Huang D., Jia Y. (2018). Maximum value projection produces better en face OCT angiograms than mean value projection. Biomed. Opt. Express.

[31] Huang L., Ding L., Zheng W. (2022). Microvascular assessment of macula, choroid, and optic disk in children with unilateral amblyopia using OCT angiography. Int. Ophthalmol..

[32] Richter G.M., Sylvester B., Chu Z., Burkemper B., Madi I., Chang R., Reznik A., Varma R., Wang R.K. (2018). Peripapillary microvasculature in the retinal nerve fiber layer in glaucoma by optical coherence tomography angiography: Focal structural and functional correlations and diagnostic performance. Clin. Ophthalmol..

[33] Lonngi M., Velez F.G., Tsui I., Davila J.P., Rahimi M., Chan C., Sarraf D., Demer J.L., Pineles S.L. (2017). Spectral-Domain Optical Coherence Tomographic Angiography in Children with Amblyopia. JAMA Ophthalmol..

[34] Demirayak B., Vural A., Onur I.U., Kaya F.S., Yigit F.U. (2019). Analysis of Macular Vessel Density and Foveal Avascular Zone Using Spectral-Domain Optical Coherence Tomography Angiography in Children with Amblyopia. J. Pediatr. Ophthalmol. Strabismus.

[35] Araki S., Miki A., Goto K., Yamashita T., Yoneda T., Haruishi K., Ieki Y., Kiryu J., Maehara G., Yaoeda K. (2019). Foveal avascular zone and macular vessel density after correction for magnification error in unilateral amblyopia using optical coherence tomography angiography. BMC Ophthalmol..

[36] Liu Y., Dong Y., Zhao K. (2017). A Meta-Analysis of Choroidal Thickness Changes in Unilateral Amblyopia. J. Ophthalmol..

[37] Baek J., Lee A., Chu M., Kang N.Y. (2019). Analysis of Choroidal Vascularity in Children with Unilateral Hyperopic Amblyopia. Sci. Rep..

[38] Daniel M.C., Dubis A.M., MacPhee B., Ibanez P., Adams G., Brookes J., Papadopoulos M., Khaw P.T., Theodorou M., Dahlmann-Noor A.H. (2019). Optical Coherence Tomography Findings After Childhood Lensectomy. Investig. Ophthalmol. Vis. Sci..

[39] Kurt R.A., Bayar S.A., Ercan Z.E., Yaman Pinarci E., Tekindal M.A., Oto S. (2021). Choroidal and Macular Thickness in Eyes with Amblyopia. Beyoglu Eye J..

[40] Zhou Y., Wang J., Jin L., Chen W., Wang Q., Chen H., Chen J., Li Z., Lin Z., Li X. (2022). Morphological characteristics of the subfoveal choroid and their association with visual acuity in postoperative patients with unilateral congenital cataracts. Ann. Transl. Med..

[41] Hui W., Xiaofeng H., Hua X., Yihan D., Yong T. (2022). Assessment of choroidal vascularity and choriocapillaris blood perfusion in Chinese preschool-age anisometropic hyperopic amblyopia children. Front. Pediatr..

[42] Sampson D.M., Gong P., An D., Menghini M., Hansen A., Mackey D.A., Sampson D.D., Chen F.K. (2017). Axial Length Variation Impacts on Superficial Retinal Vessel Density and Foveal Avascular Zone Area Measurements Using Optical Coherence Tomography Angiography. Investig. Ophthalmol. Vis. Sci..

[43] Linderman R.E., Chui T.Y.P., Langlo C.S., Curran E., Zhou D., Castanos Toral M.V., Kim J.E., Weinberg D.V., Rosen R.B., Carroll J. (2020). Assessing the Effect of Image Scaling on OCTA Vessel Density Deviation Mapping. Investig. Ophthalmol. Vis. Sci..

[44] Park K.S., Lim H.B., Shin Y.I., Park G.S., Lee W.H., Kim J.Y. (2021). Effect of axial length on peripapillary microvasculature: An optical coherence tomography angiography study. PLoS ONE.

[45] Hafner M., von Livonius B., Deschler D., Priglinger S.G., Gerhardt M.J. (2026). Quantifying segmentation sensitivity in OCTA: Device-specific profiles across three commercial platforms. PLoS ONE.

[46] Youssef M.M., Sadek S.H., Hatata R.M. (2022). Macular and Optic Nerve Microvascular Alteration in Relation to Axial Length, by Optical Coherence Tomography Angiography (OCTA). Clin. Ophthalmol..

