# Supplementary material for: Superficial Retinal Vascular Network Morphology and Sectoral RNFL Thickness in Children with a History of Bilateral Congenital Cataract Surgery: An Exploratory OCT/OCTA Study
Source: J Clin Med. 2026 Jul 7;15(13):5320. doi: 10.3390/jcm15135320 (PMC13362528; doi:10.3390/jcm15135320)
Supplement: Supplementary file 1 [file jcm-15-05320-s001.zip › jcm-4369227-supplementary.pdf]

Supplementary Table S1. Adapted Summary of Quantitative Metrics for Microvascular Network Architecture in OCTA *(Adapted from Untracht* et al., *2021* [13]).

**Metric**	**Unit**	**Description**	**Potential Biological Relevance**
Vessel area density (VAD)	%	Ratio of perfused blood vessel area (from binarized OCTA MIP image) to total image area	Reflects microvessel utilization; higher values suggest angiogenesis
Vessel length density (VLD)	%	Total vessel centerline length (from skeletonized OCTA MIP image) per total image area	Indicates possible dysfunction in oxygen/nutrient delivery; associated with angiogenesis
Vessel diameter (average/distrib.)	µm	Vessel diameters estimated via local thickness algorithm on binarized OCTA MIP	Provides insight into vascular dilation/regression; diameter distribution reflects perfusion changes
Vessel length (average/distrib.)	mm	Length of vessel segments along the centerline from skeletonized OCTA MIP	Indicates network interconnectivity and branching, reflecting tissue perfusion capabilities
Tortuosity (average/distrib.)	1	Ratio of centerline segment length to straight-line (chord) length for each vessel	Higher tortuosity suggests pathological remodeling or ischemia
Branchpoint density	nodes/mm	Number of branch nodes per unit vessel length	Reflects vascular network complexity and resistance to flow disturbances
Fractal dimension	1	Measurement of spatial complexity using box-counting method	Reflects network branching and remodeling characteristics

Abbreviations: OCTA: Optical coherence tomography angiography, MIP: Maximum intensity projection, µm: Micrometer, mm: Millimeter.
